# State Registration and Mystery

**Published:** 1919-08-16

**Authors:** 


*To the Editor of* The Hospital.

### State Registration and Mystery.

*To the Editor of* The Hospital.

Sir,—I see from a recent issue of the Journal of the blind advocates of the Central Committee's propaganda, which has accidentally dropped into my hands, that a thanksgiving service was held on July 17 at 11 Chandos Street, W. There was, according to the report, "an air of elation about the meeting," not because of anything that had been accomplished, but because "it marked another milestone passed on the long, long road." The elation is not easy to understand, inasmuch as this milestone and all the others are only monuments of failure. However, it is well to be joyful even in adversity.

It is my misfortune to have to admit that I am not keenly interested in State Registration for Nurses. My apathy is probably due to ignorance. But I have tried hard to see through the spectacles of the enthusiasts. However, a ray of sunlight from the thanksgiving service has reached me. Major Barnett, M.P., is one of the gentlemen in whose honour the service was held, and in paying her tribute Mrs. Fenwick testified that "she had never worked with any man who had taken more trouble to understand." There is apparently yet a chance for me if I take off my coat and wrap my brow with towels. I had always been under the impression that it was an easily understood problem, and that this was why Mrs. Fenwick never would in her paper write an argument setting out exactly what benefits will immediately follow legislation, and how the pilgrims will reach the promised land. As it now turns out the thing is not so simple as it looks. Major Barnett had to work very hard, and he had the advantage of personal tuition. However, I venture to suggest that a lucid demonstration might have the effect of making many converts.

With this explanation might be embodied a fascinatingly interesting chapter entitled "An upstart Council of Employers, using Matrons as their agents, and Nurses as their pawns." I quote from a hymn of hate by Mrs. Fenwick at the thanksgiving service. There are wrapt up in the title all the materials for a German spy plot, and readers will seek with interest for the sinister motive which prompts the villains. When the motive is laid bare their base designs may be frustrated.

Just one point more. The Chairman stated that "the membership of the Societies of Nurses supporting that Bill was well over 15,000." Being a doubting Thomas, but also a seeker after truth, may I inquire whether these 15,000 are trained nurses, and if there is any reason for concealing their names, addresses, and qualifications? It has all the appearance to me of one of the wonderful sea-serpent stories that we used to read about in the happy summer days long ago when there was no war.—Yours, etc.,

Ierne.

